# Acacetin Suppresses IL-1*β*-Induced Expression of Matrix Metalloproteinases in Chondrocytes and Protects against Osteoarthritis in a Mouse Model by Inhibiting NF-*κ*B Signaling Pathways

**DOI:** 10.1155/2020/2328401

**Published:** 2020-10-26

**Authors:** Jian Chen, Chen Wang, Kangmao Huang, Shuai Chen, Yan Ma

**Affiliations:** ^1^Department of Orthopaedic Surgery, Sir Run Run Shaw Hospital, Zhejiang University School of Medicine, Key Laboratory of Musculoskeletal System Degeneration and Regeneration Translational Research of Zhejiang Province, 3 East Qingchun Road, Hangzhou, Zhejiang Province, China; ^2^Department of Orthopaedic Surgery, Wuyi First People's Hospital, 2 Nanmen Street, Jinhua, Zhejiang Province, China

## Abstract

Osteoarthritis (OA) is a very common chronic joint dysfunction, and there is currently a poor understanding of its etiology and pathogenesis. Therefore, there are no active disease-modifying drugs currently available for clinical treatment. Several natural compounds have been shown to play a role in inhibiting OA progression. The present study is aimed at investigating the curative effects of acacetin, a natural flavonoid compound, against OA. Our results demonstrated that MMP-1, MMP-3, and MMP-13 were highly expressed in OA specimens. Acacetin inhibited the interleukin-1*β*- (IL-1*β*-) induced expression of MMP-1, MMP-3, and MMP-13in chondrocytes by blocking nuclear factor-*κ*B (NF-*κ*B) signaling pathways. Furthermore, we found that acacetin suppressed OA progression and inhibited the expression of MMP-1, MMP-3, and MMP-13 in ACLT-induced OA mice. Taken together, our study revealed that acacetin may serve as a potential drug for treating OA.

## 1. Introduction

Osteoarthritis (OA) is the most common age-related or posttraumatic debilitating degenerative joint dysfunction, affecting approximately 36.8% of the adult population in the USA and more than 300 million people worldwide [1]. The identified risk factors for OA include age, obesity, female gender, acute joint trauma, anatomical deformities, and hereditary predisposition [2]. Posttraumatic osteoarthritis (PTOA) accounts for approximately 12% of OA cases [3] and develops in nearly 50% of patients who experience a joint injury such as ACL tearing [4].

As a degenerative joint disorder, OA is characterized by the degeneration of cartilage, formation of osteophytes, thickening and sclerosis of subchondral bones, weakness of muscles, and inflammation in the joints [5]. Although the exact etiology of OA remains incompletely understood, it has been hypothesized that there are two main factors involved in PTOA. First, the initial injury results in irreversible structural changes that cannot be completely repaired by surgical intervention. These permanent structural changes can induce damaging mechanical forces and place abnormal loads on the cartilage [6]. Second, proinflammatory cytokines induced by the injury cause degradation of the extracellular matrix (ECM) and apoptosis in chondrocytes by activating reactive oxygen species and upregulating the activity of proteases such as matrix metalloproteinases (MMPs) and aggrecanases [7]. Among the proinflammatory cytokines, interleukin-1*β* (IL-1*β*) and tumor necrosis factor-*α* (TNF-*α*) are the major proinflammatory cytokines involved in OA development; they are produced by chondrocytes, mononuclear cells, osteoblasts, and synovial tissues [8]. Ultimately, the reduced mechanical strength and ECM breakdown contribute to rapid disease progression [9].

Despite the prodigious burden of this disease worldwide, no effective disease-modifying drugs have been developed to prevent OA progression [10]. Furthermore, early diagnosis and developmental-stage identification of OA also remain challenging due to the high prevalence of asymptomatic patients [11]. Therefore, understanding the mechanisms controlling chondrocyte degeneration and administering drugs after trauma or in early stages may be pivotal for preventing OA progression. Recent studies have reported several natural compounds that possess anti-inflammatory functions in chondrocytes [12–14]. Acacetin (5,7-dihydroxy-4-methoxyflavone) is a natural flavonoid compound with anti-inflammatory, antiperoxidative, and anticancer activities [15, 16]. However, it is unclear whether acacetin can inhibit the expression of catabolic enzymes to prevent cartilage degeneration. In this study, we investigated the protective effect of acacetin on cartilage *in vitro* and *in vivo*.

## 2. Materials and Methods

### 2.1. Human Specimens

This study was approved by the Ethics Committee of Sir Run Run Shaw Hospital and carried out in accordance with the World Medical Association Declaration of Helsinki. All patients signed informed consent. Samples of control tissues were obtained from patients with femoral neck fracture without a history of OA undergoing total hip arthroplasty (*n* = 3). Femoral neck fracture was diagnosed based on X-rays and symptoms. Samples of OA tissues were also obtained from end-stage symptomatic hip OA patients treated with total hip arthroplasty. OA was diagnosed according to X-rays and clinical symptoms.

### 2.2. RNA Extraction and Quantitative PCR (qPCR)

Total RNA was extracted from cartilage samples or chondrocytes using TRIzol reagent and Ultrapure RNA Kit (KWBIO, Shanghai, China). cDNA was synthesized from 1 *μ*g RNA using reverse transcription reagent (KWBIO, Shanghai, China) according to the manufacturer's instructions. ABI Prism 7500 (Applied Biosystems, Thermo Fisher Scientific, Waltham, MA, USA) was used to detect mRNA expression by qPCR. The reaction system consisted of 10 *μ*L UltraSYBR Mixture (KWBIO, Shanghai, China), 8 *μ*L ddH_2_O, 1 *μ*L cDNA, and 1 *μ*L primers (Sangon Biotech, Shanghai, China). The primer sequences are shown in Table 1. The cycling program was as follows: 95°C for 10 min, followed by 40 cycles of denaturation at 95°C for 15 s, and annealing/extension at 60°C for 1 min. The relative expression of the target genes was calculated using the 2^-*ΔΔ*CT^ method. Data were normalized against *GAPDH* and presented as the mean ± standard deviation (SD).

### 2.3. Cell Transfection

Chondrocytes were cultured in 6-well plates with appropriate cells for 24 h. pcDNA3.1 (vector control) or pcDNA3.1-IKK*β* (Vigene Biosciences, Shanghai, China) were transfected into chondrocytes using Lipofectamine 3000 (Invitrogen, Carlsbad, CA, USA) according to the manufacturer's instructions. Twenty-four hours after transfection, the cells were used for the following experiments.

### 2.4. Western Blotting

To determine the effect of acacetin (Sigma-Aldrich, St. Louis, USA) on the expression of MMP-1 (ab137332), MMP-3 (sc6839), and MMP-13 (sc30073) induced by IL-1*β* in chondrocytes, chondrocytes were seeded in six-well plates at a density of 5 × 10^5^ cells per well. The cells were treated with acacetin (6.25 or 3.125 *μ*M) with or without IL-1*β* (10 ng/mL) for 48 h. Total protein was extracted using RIPA lysis buffer supplemented with protease inhibitor (Sigma-Aldrich, St. Louis, USA). Lysates were centrifuged at 12,000 × *g* and 4°C for 15 min. Next, the supernatants were collected, and the protein concentrations were analyzed using a bicinchoninic acid (BCA) kit (Thermo Fisher Scientific, Waltham, MA, USA).

We further investigated which signaling pathways were targeted by acacetin. The seeded chondrocytes were pretreated with acacetin (6.25 *μ*M) for 4 h and then stimulated with IL-1*β* (10 ng/mL) for another 0, 10, or 30 min. Total protein was extracted as described above. Western blotting was then used to quantify phosphor-P38 (Thr180/Tyr182, sc-17852), P38 (sc-535), phospho-ERK (Thr202/Tyr204, sc-16981), ERK (sc-292838), phospho-JNK (Thr183/Tyr185, sc-6254), JNK (sc-572), phospho-I*κ*B-*α* (sc-8404), I*κ*B-*α* (sc-847), IKK*α* (ab32041), IKK*β* (ab124957), and phospho-IKK*α*/*β* (Abcam, 16A6).

To confirm the effect of acacetin on I*κ*B-*α* signaling, we overexpressed IKK*β* through transfection and treated the cells first for 4 h with or without acacetin (6.25 *μ*M) and then with IL-1*β* (10 ng/mL) for 30 min. Total protein was extracted as described above. Western blotting was then used to quantify IKK*β*, phospho-IKK*α*/*β*, and phospho-I*κ*B-*α*. In addition, cells overexpressing IKK*β* were treated for 4 h with or without acacetin (6.25 *μ*M) and then with IL-1*β* (10 ng/mL) for 48 h. Total protein was extracted as described above. Western blotting was then used to quantify MMP-1, MMP-3, and MMP-13.

Equal volumes (10 *μ*L) of each sample were resolved using 8% or 10% SDS-PAGE and then transferred to PVDF membranes by electroblotting. The PVDF membranes were blocked with 5% (*v*/*v*) skim milk dissolved in TBST at room temperature for 1 h and then incubated with the indicated primary antibodies at 4°C overnight. After being washed three times with TBST, membranes were incubated with horseradish peroxidase-conjugated secondary antibody (Beyotime Institute of Biotechnology Inc., Jiangsu, China) for 1 h at room temperature. After being washed further three times with TBST, proteins were exposed to electrochemical luminescence reagent and visualized on an Odyssey infrared imaging system. ImageJ software was used to quantify the relative intensity of the protein bands.

### 2.5. Immunohistochemistry and Histological Analysis

Human cartilage samples and murine knee joints were fixed with 4% (*v*/*v*) paraformaldehyde for 24 h. After decalcification in 10% EDTA at room temperature for 4 weeks, knee joint samples were dehydrated using a graded ethanol series and then embedded in paraffin. Samples were sliced into 4 *μ*m sections at five-slide intervals and stained with Safranin O/fast green for morphometric examination. The Osteoarthritis Research Society International (OARSI) histopathology grading and staging system was used to grade cartilage tissue degeneration [17]. For immunostaining, slides were incubated with antibodies targeting MMP-1, MMP-3, and MMP-13 at 4°C overnight. After being washed with PBS, slides were incubated with secondary antibody for 2 h at room temperature. Positively stained cells on the articular surface were counted and given as a percentage.

### 2.6. Cell Culture

Primary murine articular cartilage samples were harvested from the tibial plateaus of C57B/6 mice (purchased from Shanghai SLAC Laboratory Animal Co., Ltd. (Shanghai, China)) at 5 days postnatal. Primary human articular cartilage samples were harvested from control cartilage tissue. Both human and murine cartilage specimens were minced and washed with PBS three times. The finely divided cartilage tissue was digested with 0.25% collagenase in DMEM at 37°C overnight. Chondrocytes were obtained by centrifugation and cultured in DMEM with 10% (*v*/*v*) fetal bovine serum (FBS) at 37°Cwith 5% CO_2_. Cells cultured up to the third passage were used for further experiments.

### 2.7. Cell Viability Assay

The cytotoxicity of acacetin was evaluated using cell counting kit 8 (CCK-8) assay (Sigma-Aldrich, St. Louis, USA). Primary human chondrocytes were seeded in 96-well plates at a density of 5 × 10^3^ cells per well. The cells were treated with graded concentrations of acacetin (0, 3.125, 6.25, 12.5, 25, 50, 100, and 200 *μ*M) for 48 h. Following this, 10 *μ*L CCK-8 buffer was added to each well. Following incubation for 2 h at 37°C, absorbance at 450 nm (650 nm reference) was measured using ELX800 (Bio-Tek, Winooski, VT, USA). Cell viability was calculated based on six independent experiments.

### 2.8. Cell Immunofluorescence

To assess whether acacetin inhibited IL-1*β*-induced MMP expression, articular chondrocytes were seeded in 12-well plates at a density of 2 × 10^5^ cells per well. Chondrocytes were treated with acacetin (3.125, 6.25 *μ*M) with or without IL-1*β* (10 ng/mL) for 48 h. The chondrocytes were fixed with 4% paraformaldehyde and permeabilized with 0.2% Triton X-100 for 10 min, and cells were incubated with antibodies targeting MMP-1, MMP-3, and MMP-13 at 4°C overnight. Next, cells were washed and incubated with fluorescein isothiocyanate- (FITC-) conjugated goat anti-rabbit secondary antibodies at room temperature for 1 h. Cells were then counterstained with DAPI (Life Technologies, Carlsbad, CA, USA) to visualize nuclear DNA and examined using an Olympus confocal imaging system (FV100; Olympus, Tokyo, Japan).

To confirm the effect of acacetin on the nuclear factor-*κ*B (NF-*κ*B) pathway, chondrocytes were stimulated with IL-1*β* (10 ng/mL) for another 30 min after acacetin was administered to cells for 4 h. Immunofluorescence staining for P65 (D14E12, CST) was also performed as described above.

### 2.9. Anterior Cruciate Ligament Transection- (ACLT-) Induced OA and Acacetin Treatment in Mice

All C57BL/6 mice, aged 8 weeks, were purchased from Shanghai SLAC Laboratory Animal Co., Ltd. (Shanghai, China). C57BL/6 mice (*n* = 24) were divided equally into four groups. Mice in group I received a sham knee surgery. The other three groups received ACLT surgery as described in a previous study [18]. All experimental procedures were performed in accordance with the Guide for the Care and Use of Laboratory Animals, as adopted and promulgated by the United States National Institutes of Health, as well as in accordance with the Guide of the Animal Care Committee of Zhejiang University. The animal study was performed in the animal experimental center of Sir Run Run Shaw Hospital. After 12 days, group II began to receive intra-articular injections of normal saline twice a week. Group III and group IV received intra-articular injections of L-acacetin (low dose, 3.125 *μ*M) and H-acacetin (high dose, 6.25 *μ*M), respectively, at the same frequency. After 6 weeks, the distal femurs of mice were harvested for histological and immunohistochemical analysis.

### 2.10. Statistical Analysis

Statistical analyses were performed using SPSS 22.0 (SPSS, Chicago, IL, USA). Data are represented as the mean ± SEM. The significance of differences between two groups was determined using an independent-sample *t*-test. The significance of differences between three or more groups was analyzed using one-way ANOVA and Tukey's post hoc test or Student's *t*-test. A value of *P* < 0.05 was considered statistically significant.

## 3. Results

### 3.1. Expression of MMP-1, MMP-3, and MMP-13 in Human Cartilages

**Figure 1:**
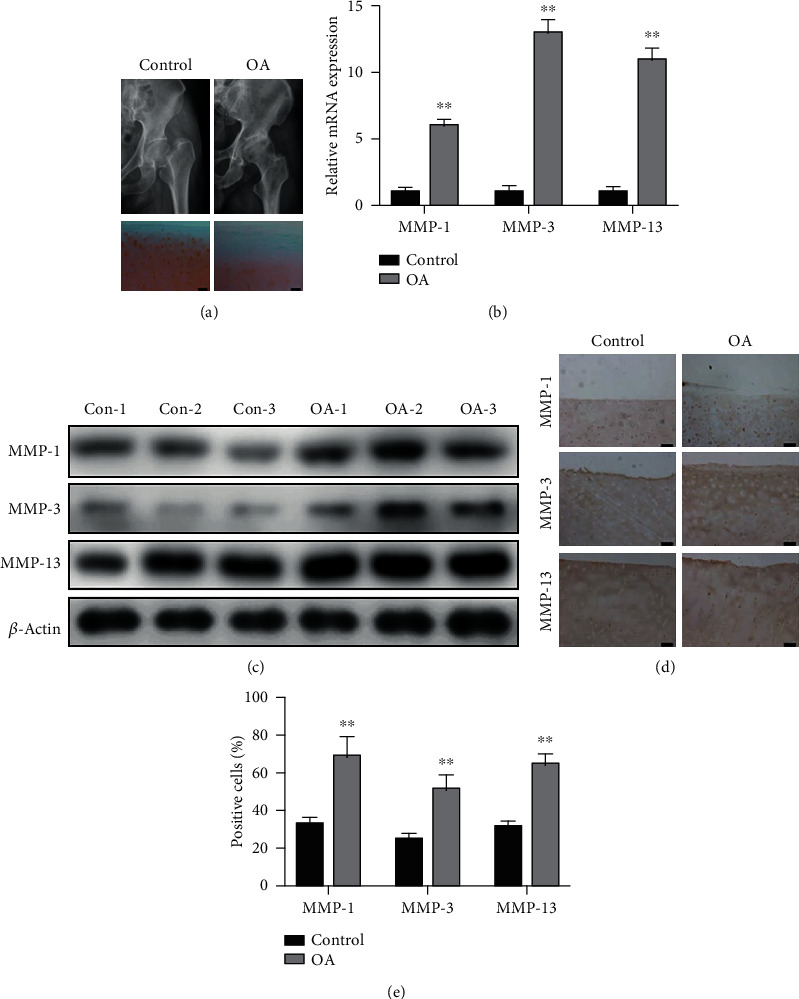
Safranin O/fast green staining and expression of matrix metalloproteinases (MMPs) in cartilage samples from controls and patients with OA. (a) X-rays of hip joints and Safranin O/fast green staining of control and OA cartilage samples. Scale bar = 100 nm. (b) The relative mRNA expression of *MMP-1*, *MMP-3*, and *MMP-13* in control and OA cartilage tissues. (c) The protein expression of MMP-1, MMP-3, and MMP-13 in control and OA cartilage tissues. (d) Immunohistochemical staining of cartilage tissues from OA and control patients for MMP-1, MMP-3, and MMP-13. (e) Percentages of MMP-1-, MMP-3-, and MMP-13-positive cells in control and OA cartilage samples. *N* = 3, ^∗^*P* < 0.05, and ^∗∗^*P* < 0.01. Scale bar = 50 *μ*m.

To evaluate the expression of MMP-1, MMP-3, and MMP-13 in OA, we harvested cartilage specimens from fracture and OA patients who were diagnosed via X-ray. Human cartilage tissues from the femoral heads of fracture patients displayed more Safranin O-positive proteoglycans than did those from OA patients (Figure 1(a)). The relative mRNA and protein expression levels of MMP-1, MMP-3, and MMP-13 in cartilage tissues were determined using qPCR and western blotting, respectively. Figures 1(b) and 1(c) show that the expression levels of MMP-1, MMP-3, and MMP-13 were higher in OA tissues than in control tissues. Furthermore, the protein levels of the MMPs were also evaluated using immunohistochemistry. The OA samples showed more MMP-1-, MMP-3-, and MMP-13-positive cells than the control samples (Figures 1(d) and 1(e)), consistent with the results of qPCR and western blotting.

### 3.2. Acacetin Inhibits IL-1*β*-Induced Expression of MMPs in Chondrocytes

**Figure 2:**
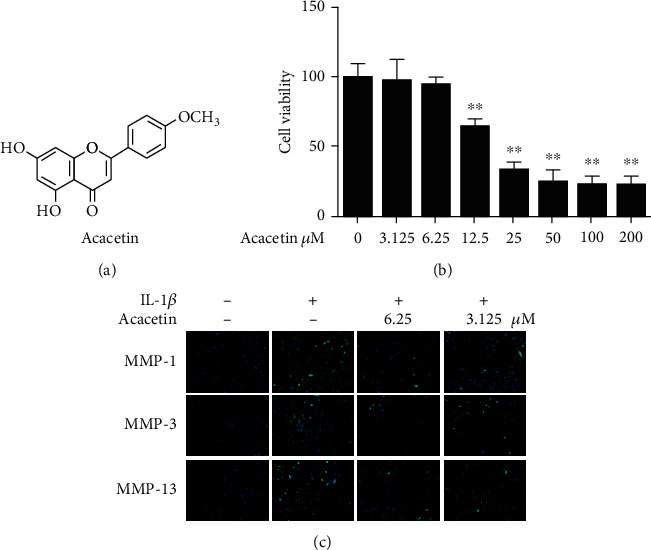
Cytotoxicity and MMP inhibitory effect of acacetin in human primary chondrocytes. (a) Chemical structural formula of acacetin. (b) Viability of chondrocytes treated with acacetin (0–200 *μ*M) for 48 h. (c) Chondrocytes were treated with acacetin (H-acacetin, 6.25 *μ*M; L-acacetin, 3.125 *μ*M) and IL-1*β* (10 ng/mL) for 24 h. Immunofluorescence showed the expression of MMP-1, MMP-3 and MMP-13 in the cytoplasm of chondrocytes. *N* = 3, ^∗^*P* < 0.05, and ^∗∗^*P* < 0.01.

Next, we assessed the influence of acacetin on IL-1*β*-stimulated chondrocytes. We first investigated the cytotoxicity of acacetin in human chondrocytes using a CCK-8 assay. No detectable cytotoxic effects of acacetin were observed at concentrations below 6.25 *μ*M for 48 h (Figures 2(a) and 2(b)). Thus, concentrations of 6.25 *μ*M (H-acacetin) and 3.125 *μ*M (L-acacetin) were adopted for further experiments. As shown by the immunofluorescence results (Figure 2(c)), the upregulation of MMP-1, MMP-3, and MMP-13 induced byIL-1*β* was inhibited by acacetin in human chondrocytes in a dose-dependent manner.

**Figure 3:**
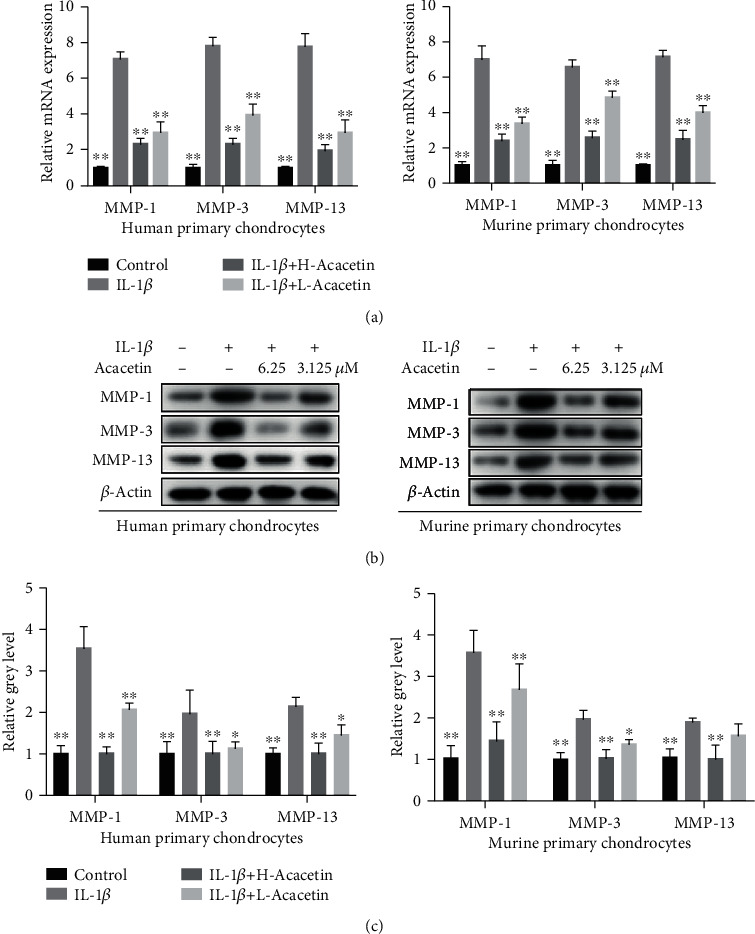
Acacetin inhibits the expression of MMP-1, MMP-3, and MMP-13 in IL-1*β*-stimulated human and murine primary chondrocytes. (a) The mRNA expression levels of *MMP-1*, *MMP-3*, and *MMP-13* in chondrocytes treated with IL-1*β* (10 ng/mL) and acacetin (H-acacetin, 6.25 *μ*M; L-acacetin, 3.125 *μ*M) for 24 h. (b) Western blot showing MMP-1, MMP-3, and MMP-13 activity in chondrocytes treated with IL-1*β* (10 ng/mL) and H- or L-acacetin for 48 h. (c) Relative intensity of protein bands normalized to *β*-actin (*n* = 6). *N* = 3, ^∗^*P* < 0.05, and ^∗∗^*P* < 0.01.

To verify the impact of acacetin on IL-1*β*-treated chondrocytes, we examined the mRNA and protein expression levels of MMP-1, MMP-3, and MMP-13 in human and murine primary chondrocytes. Consistent with the results of immunofluorescence, the IL-1*β*-induced increase in the expression of MMP-1, MMP-3, and MMP-13 was significantly repressed by both H-acacetin and L-acacetin (Figure 3). Overall, these results indicated that acacetin potentially slowed the degeneration process in chondrocytes by repressing the expression of MMP-1, MMP-3, and MMP-13 induced by IL-1*β*.

### 3.3. Acacetin Inhibits IL-1*β*-Induced Activation of Nuclear Factor- (NF-) *κ*B Pathways

**Figure 4:**
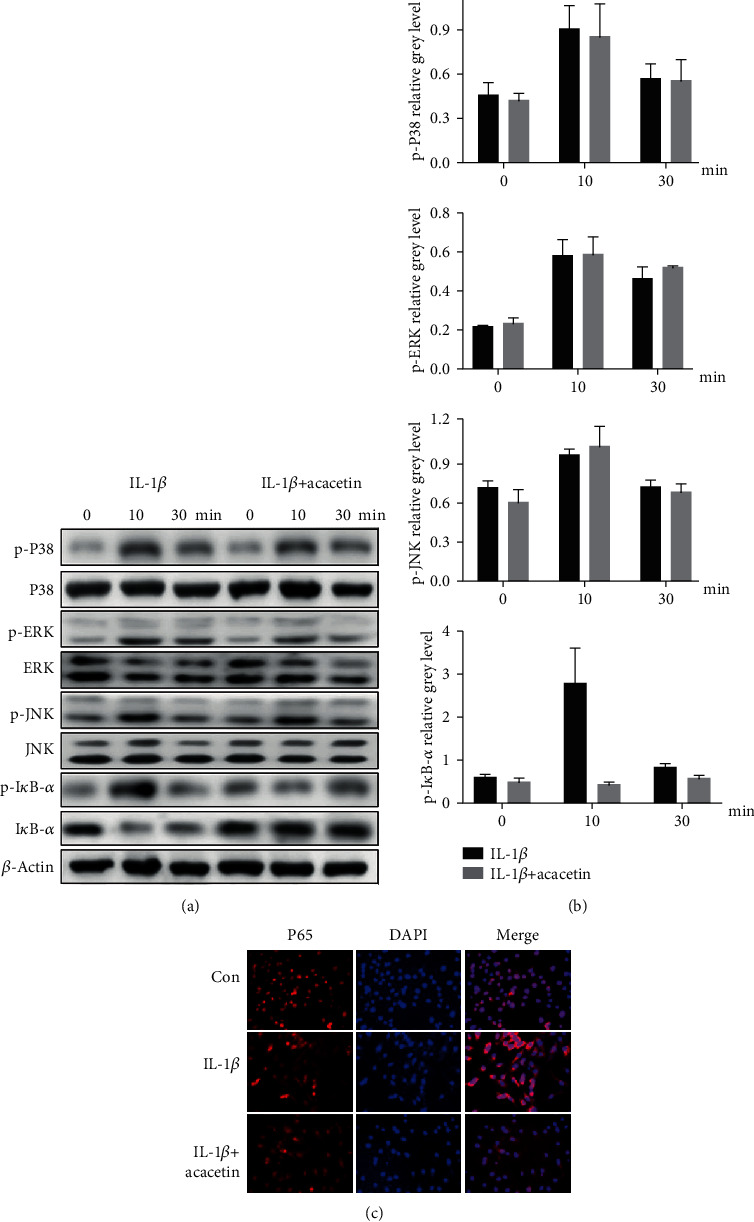
Acacetin inhibits IL-1*β*-induced activation of nuclear factor- (NF-) *κ*B pathways. (a) Chondrocytes were pretreated with acacetin (6.25 *μ*M) for 4 h and then incubated with IL-1*β* (10 ng/mL) for the indicated time periods (0, 10, and 30 min). The protein levels of p-P38, P38, p-extracellular signal-regulated kinase (ERK), ERK, p-c-Jun N-terminal kinase (JNK), JNK, p-inhibitor of NF-*κ*B-*α* (I*κ*B-*α*), and *β*-actin were determined by western blotting. (b) The quantification of relative protein expression normalized to *β*-actin. (c) When IL-1*β*-stimulated chondrocytes were treated with acacetin (6.25 *μ*M), the localization of P65 changed from the nucleus to the cytoplasm. *N* = 3, ^∗^*P* < 0.05, and ^∗∗^*P* < 0.01.

We analyzed the mitogen-activated protein kinase (MAPK) and NF-*κΒ* pathways to ascertain the mechanisms by which acacetin represses the activation of IL-1*β*-induced catabolic proteases. Consistent with previous studies, IL-1*β* significantly stimulated the activation of the NF-*κΒ* and MAPK pathways, including factors such as extracellular signal-regulated kinase (ERK), P38, and c-Jun N-terminal kinase (JNK). However, no MAPK pathway-related factors were affected. The inhibited degradation of inhibitor of NF-*κ*B-*α* (I*κ*B-*α*) caused by acacetin suggested that the activation of the NF-*κΒ* pathway was suppressed (Figures 4(a) and 4(b)). To validate that the NF-*κΒ* pathway was involved in the acacetin-induced protective effect, we investigated the localization of P65 in chondrocytes. The results showed that P65 nuclear translocation was increased after IL-1*β* treatment, but decreased after treatment with acacetin (Figure 4(c)).

### 3.4. Acacetin Inhibited IL-1*β*-Induced MMP Expression by Targeting NF-*κ*B Pathways

**Figure 5:**
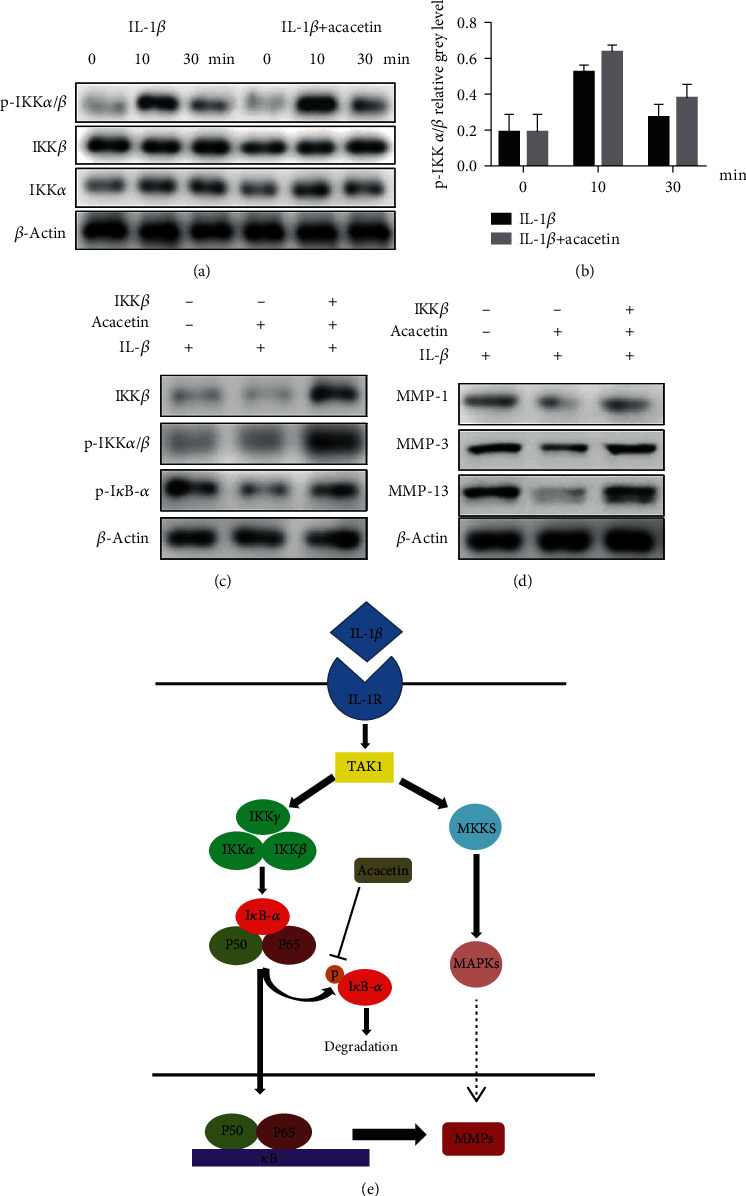
Acacetin inhibited IL-1*β*-induced MMP expression by targeting NF-*κ*B pathways. (a) Chondrocytes were pretreated with acacetin (6.25 *μ*M) for 4 h and then stimulated with IL-1*β* (10 ng/mL) for the indicated time periods (0, 10, and 30 min). Protein levels of p-I*κ*B kinase (IKK)*α*/*β*, IKK*α* and IKK*β*, were evaluated by western blotting. (b) The relative intensity of protein bands. (c) IKK*β*-overexpressing chondrocytes were treated for 4 h with or without acacetin (6.25 *μ*M) and then with IL-1*β* (10 ng/mL) for 30 min. Western blotting was then used to quantify IKK*β*, phospho-IKK*α*/*β*, and phospho-I*κ*B-*α*. (d) IKK*β*-overexpressing chondrocytes were treated for 4 h with or without acacetin (6.25 *μ*M) and then with IL-1*β* (10 ng/mL) for 48 h. Western blotting was then used to quantify MMP-1, MMP-3, and MMP-13. (e) Schematic of the proposed mechanism showing that acacetin inhibits MMP expression in chondrocytes by blocking P38 and NF-*κ*B pathways. *N* = 3, ^∗^*P* < 0.05, and ^∗∗^*P* < 0.01.

To further examine the upstream regulators of the NF-*κΒ* pathways, we studied the expression of p-I*κ*B kinase (IKK)*α*/*β*, which play important roles in IL-1*β*-related signaling pathways. When IL-1*β*-treated cells were incubated with acacetin, no significant differences in the expression of IKK*α*, IKK*β*, or p-IKK*α*/*β* were detected (Figures 5(a) and 5(b)). These results indicate that IL-1*β*-induced activation of NF-*κΒ* pathway and expression of MMPs could be suppressed by acacetin. However, overexpression of IKK*β* efficiently abrogated the influences caused by acacetin (Figures 5(c) and 5(d)). We concluded that acacetin suppressed the IL-1*β*-induced expression of MMP-1, MMP-3, and MMP-13 in chondrocytes by targeting NF-*κΒ* signaling pathways (Figure 5(e)).

### 3.5. Acacetin Prevents Cartilage Degeneration in ACLT-Induced OA

**Figure 6:**
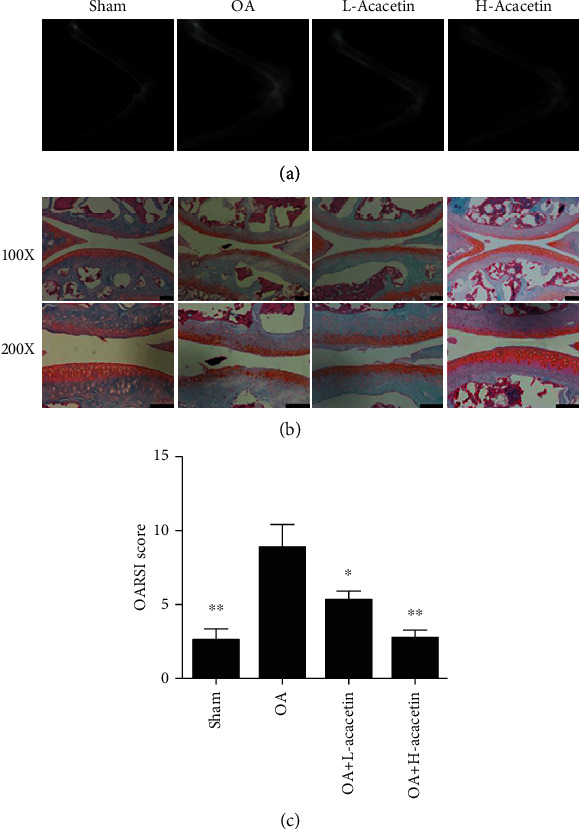
Acacetin prevents progression of anterior cruciate ligament transection (ACLT)-induced OA. (a) X-rays of the knee joints from mice in the sham, OA, H-acacetin, and L-acacetin groups. (b) Safranin O/fast green staining of cartilage samples from knee joints in the four groups at 6 weeks after ACLT surgery. (c) Osteoarthritis Research Society International (OARSI) scores of knee joint sections from the four groups. *n* = 6, ^∗^*P* < 0.05, and ^∗∗^*P* < 0.01. Scale bar = 50 *μ*m.

**Figure 7:**
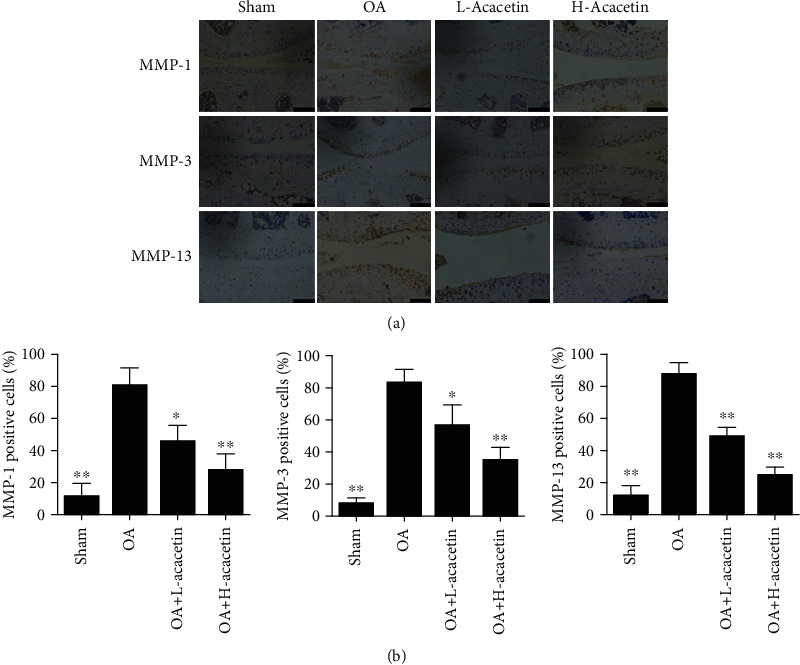
Acacetin inhibits MMP expression *in vivo*. (a) Immunohistochemical analysis of cartilage samples from knee joints for MMP-1, MMP-3, and MMP-13 at 6 weeks after ACLT surgery in mice in the sham, OA, H-acacetin, and L-acacetin groups (*n* = 6 per group). (b) Percentages of immune-positive cells in each group. *n* = 6, ^∗^*P* < 0.05, and ^∗∗^*P* < 0.01. Scale bar = 50 *μ*m.

To determine whether acacetin exerted a protective effect *in vivo*, we constructed an OA model by performing ACLT in mice. L-acacetin (3.125 *μ*M) and H-acacetin (6.25 *μ*M) were administered by intra-articular injection after injury. After 6 weeks, the knee joints were collected for X-ray radiographs and Safranin O staining. The OA group showed more severe joint space narrowing and lower proteoglycan levels than the sham group. However, the narrowed joint space and reduced proteoglycan levels were reversed by acacetin in a dose-dependent manner (Figures 6(a) and 6(b)). The OARSI scores indicated that both L-acacetin and H-acacetin prevented cartilage degradation (Figure 6(c)). Furthermore, the expression of catabolic proteases, including MMP-1, MMP-3, and MMP-13 also decreased when acacetin was injected (Figure 7). In summary, these results reveal that acacetin halted OA progression *in vivo*.

## 4. Discussion

After a joint injury, it usually takes 10–15 years for OA to develop [9]. Consequently, effective pharmacological therapies are necessary to halt OA development to delay or prevent the need for joint replacement surgery. Acacetin has been demonstrated to have anti-inflammatory activity in lipopolysaccharide-induced acute lung injuries, cardiomyocytes, and neuronal diseases [19, 20]. We considered that acacetin might have an anti-inflammatory effect on chondrocytes. In this study, we found that acacetin reduced the IL-1*β*-induced inflammatory reaction and expression of catabolic enzymes in chondrocytes, showing that it may be a beneficial drug for OA treatment.

The MMP family comprises zinc-dependent enzymes that are involved in tissue remodeling and protein degradation in the ECM [21]. MMP-1 and MMP-13 are collagenases that play a pivotal role in hydrolyzing fibrillar collagen types I, II, and III [22]. MMP-3 is a stromelysin that participates in pro-MMP activation [23]. Our results showed an increased expression of MMP-1, MMP-3, and MMP-13, as well as a reduced expression of proteoglycans, in samples from patients with OA compared to levels in samples from patients with fracture. In particular, MMP-13 has received a major attention because of its central position in the cartilage degradation network and its efficiency in collagen II degradation [24]. For instance, the development of OA was shown to be repressed by preventing proteoglycan loss and structural degeneration in MMP-13-knockout mice using a medial meniscal destabilization model [25]. Furthermore, MMP-13 is known to be a major factor in early onset OA [26, 27]. Our results demonstrated that acacetin decreased the expression of MMP-13 in chondrocytes, which suggests that it may obstruct the progression of OA in early stages.

IL-1*β* elevation has been commonly detected in OA cartilage, subchondral bone, synovial fluid, and synovial membrane [8, 28]. As a driver of OA progression, IL-1*β* specifically binds to receptors on the cell membrane, activates the intracellular MAPK and NF-*κ*B signal transduction pathways, and, through a series of signal transductions, causes an increase in MMPs [29–31]. Therefore, developing anti-inflammatory therapeutics targeting IL-1*β* signaling is a promising strategy [32]. In addition to the association of protein synthesis signaling with ECM degradation, MAPK signaling pathways are also involved in chondrocyte apoptosis, calcification, and proliferation signal transduction [33]. Previous studies have demonstrated that the inhibition of MAPK and NF-*κ*B activity reduces the expression of inflammatory mediators in chondrocytes [34–36]. In this study, we found that acacetin could inhibit the expression of MMPs through the NF-*κ*B signaling pathway. It was reported that acacetin inhibits the phosphorylation of P38 and JNK and reduces the expression of MMP-1, MMP-3, and MMP-13 in IL-1*β*-induced fibroblast-like synoviocytes [37]. However, the same study showed that acacetin had no effect on the DNA-binding activity of NF-*κ*B [38], although this was inhibited by acacetin in other studies [39, 40]. Different cell types and exposure times may have contributed to this discrepancy. We also detected the upstream signals in IL-1*β*-induced chondrocytes. Interestingly, the upstream pathways of NF-*κ*B were not influenced by acacetin. In addition, overexpression of IKK*β* efficiently abrogated the influences caused by acacetin. These findings suggest that acacetin exerts anti-inflammatory effects mainly through the inhibition of NF-*κ*B pathways.

There were several limitations to our study. First, we investigated the effects of acacetin on chondrocytes alone. Although subchondral bone and synovial membranes participate in the pathological progression of OA, we did not investigate how other joint tissues were influenced by acacetin treatment. Furthermore, different delivery systems may affect the efficiency of acacetin. For instance, liposomes [41], avidin nanocarriers [42], and polyethylene-glycol-modified single-walled carbon nanotubes [43] have been investigated as alternative drug delivery methods; increasing the penetration and retention time of acacetin may help to slow the progression of OA.

## 5. Conclusions

MMPs play an important role in tissue remodeling and protein degradation in the ECM. Our study showed that acacetin suppressed the expression of MMP-1, MMP-3, and MMP-13 by inhibiting NF-*κ*B pathways and exerted a protective effect on cartilage, indicating its potential value in the treatment of OA.

## Figures and Tables

**Table 1:** PCR primers.

Gene	Strand	Primer sequences	Origin
*GAPDH*	Forward	5′-AGGGCCCTGACAACTCTTTT-3′	Human
	Reverse	5′-AGGGGTCTACATGGCAACTG-3′	
*MMP-1*	Forward	5′-CAAGTGCTGTGGCACTGTTT-3′	Human
	Reverse	5′-ACTTGTCCCAGCATTGAACC-3′	
*MMP-3*	Forward	5′-CTGGGAAAATCAGCCATTGT-3′	Human
	Reverse	5′-AGGTTCTGGAGGGACAGGTT-3′	
*MMP-13*	Forward	5′-AACATCCAAAAACGCCAGAC-3′	Human
	Reverse	5′-TTGGCAATATGCAGAAGCAG-3′	
*GAPDH*	Forward	5′-ACCCAGAAGACTGTGGATGG-3′	Mouse
	Reverse	5′-CACATTGGGGGTAGGAACAC-3′	
*MMP-1a*	Forward	5′-CTAAGGCAGGAGGATTGCTG-3′	Mouse
	Reverse	5′-TGCGAAGGGCTTAGTGTCTT-3′	
*MMP-3*	Forward	5′-CTCAGAGGAGCAAGGGTTTG-3′	Mouse
	Reverse	5′-CAACTGCGAAGATCCACTGA-3′	
*MMP-13*	Forward	5′-GAGCCACAGATGAGCACAGA-3′	Mouse
	Reverse	5′-ATGTAAGGCCACCTCCACTG-3′	

## Data Availability

The data used to support the findings of this study are included within the article.
